# Capturing postural blood pressure dynamics with near-infrared spectroscopy-measured cerebral oxygenation

**DOI:** 10.1007/s11357-023-00791-9

**Published:** 2023-04-12

**Authors:** Marjolein Klop, Rianne A. A. de Heus, Andrea B. Maier, Anne van Alphen, Marianne J. Floor-Westerdijk, Mathijs Bronkhorst, René J. F. Melis, Carel G. M. Meskers, Jurgen A. H. R. Claassen, Richard J. A. van Wezel

**Affiliations:** 1 Department of Biophysics, Donders Institute for Brain, Cognition and Behaviour, Radboud University, Nijmegen, the Netherlands; 2 Department of Geriatric Medicine, Radboud University Medical Center, Nijmegen, the Netherlands; 3 Department of Primary and Community Care, Radboud University Medical Center, Nijmegen, the Netherlands; 4 Department of Human Movement Sciences, @AgeAmsterdam, Amsterdam Movement Sciences, Vrije Universiteit Amsterdam, Amsterdam, the Netherlands; 5 Healthy Longevity Translational Research Program, Yong Loo Lin School of Medicine, National University of Singapore, Singapore, Singapore; 6 Centre for Healthy Longevity, @AgeSingapore, National University Health System, Singapore, Singapore; 7 Artinis Medical Systems, Elst, the Netherlands; 8 Department of Rehabilitation Medicine, Amsterdam Movement Sciences, Amsterdam University Medical Center, Amsterdam, the Netherlands; 9 Department of Cardiovascular Sciences, University of Leicester, Leicester, UK; 10 Department of Biomedical Signals and Systems, Technical Medical Centre, University of Twente, Enschede, the Netherlands

**Keywords:** Near-infrared spectroscopy, Orthostatic hypotension, Blood pressure, Cerebral oxygenation, Aging

## Abstract

Orthostatic hypotension (OH) is highly prevalent in older adults and associated with dizziness, falls, lower physical and cognitive function, cardiovascular disease, and mortality. OH is currently diagnosed in a clinical setting with single-time point cuff measurements. Continuous blood pressure (BP) devices can measure OH dynamics but cannot be used for daily life monitoring. Near-infrared spectroscopy (NIRS) has potential diagnostic value in measuring cerebral oxygenation continuously over a longer time period, but this needs further validation. This study aimed to compare NIRS-measured (cerebral) oxygenation with continuous BP and transcranial Doppler-measured cerebral blood velocity (CBv) during postural changes. This cross-sectional study included 41 participants between 20 and 88 years old. BP, CBv, and cerebral (long channels) and superficial (short channels) oxygenated hemoglobin (O_2_Hb) were measured continuously during various postural changes. Pearson correlations between BP, CBv, and O_2_Hb were calculated over curves and specific characteristics (maximum drop amplitude and recovery). BP and O_2_Hb only showed good curve-based correlations (0.58–0.75) in the initial 30 s after standing up. Early (30–40 s) and 1-min BP recovery associated significantly with O_2_Hb, but no consistent associations were found for maximum drop amplitude and late (60–175 s) recovery values. Associations between CBv and O_2_Hb were poor, but stronger for long-channel than short-channel measurements. BP associated well with NIRS-measured O_2_Hb in the first 30 s after postural change. Stronger associations for CBv with long-channel O_2_Hb suggest that long-channel NIRS specifically reflects cerebral blood flow during postural transitions, necessary to better understand the consequences of OH such as intolerance symptoms.

**Supplementary Information:**

The online version contains supplementary material available at 10.1007/s11357-023-00791-9.

## Introduction

Orthostatic hypotension (OH) is classically defined as a prolonged blood pressure (BP) drop upon standing of at least 20 mmHg systolic and/or 10 mmHg diastolic that occurs within the first 3 min after standing up [1]. OH is a prevalent condition in older adults, varying from 6% in the general population [2] up to 22% in people aged 60 years or older [3]. OH can have detrimental clinical outcomes, such as falls [4] and worse physical performance [5]. People who have OH also have higher risks of cognitive impairment [6], cardiovascular disease [7], and mortality [8]. Advancing current diagnosis and monitoring requires looking beyond the classic definition of OH, towards capturing the dynamics of posture-related changes in BP and their effects on cerebral perfusion pressure, cerebral blood flow (CBF), and cerebral oxygenation under daily life conditions [9–12]. Current clinical tools to measure OH are mainly intermittent and in some specialized centers non-invasive continuous BP measurements, but these are not suited for diagnosing or monitoring OH in daily life conditions. Another limitation is that measurements restricted to BP do not capture the effects of postural change on cerebral perfusion, while these are probably responsible for most OH-related symptoms [13]. Non-invasive, ambulant measurements of oxygenated (O_2_Hb), deoxygenated hemoglobin (HHb) and total hemoglobin (tHb) in cerebral frontal lobe tissue using near-infrared spectroscopy (NIRS) may be an alternative, but need further validation [14].

Previous work has shown how O_2_Hb and HHb are affected by postural changes from supine, sitting, or squatting to standing. Upon standing, the relative O_2_Hb concentration drops, followed by recovery towards a new equilibrium, which is somewhat lower than the baseline sitting or supine O_2_Hb. These effects were observed in healthy young and older adults. Simultaneously, HHb increases after standing up and recovers to a value above baseline [15–18]. Within the first 30 s of standing, Pearson correlation coefficients between O_2_Hb and BP measured by volume-clamp photoplethysmography ranged from 0.66 to 0.94 depending on the type of postural change [17]. Associations between O_2_Hb and BP in the remaining of the 3 min after standing have not been described.

NIRS can encompass different inter-optode distances of <1 cm (short channels) and 3–4 cm (long channels). Long-channel optodes are placed at such a distance that light is assumed to travel deep enough to penetrate through cerebral tissue. Short channels, however, are assumed to be restricted to measuring extracranial tissue and are therefore often used in functional NIRS (fNIRS) research to distinguish intra- from extracranial oxygenation responses [19, 20]. When studying OH, short-channel NIRS could in theory be used to reflect tissue perfusion changes due to changes in BP, unaffected by autoregulation, whereas long-channel NIRS would also reflect CBF changes which are modified by autoregulation [21]. To explore this, cerebral hemodynamic responses to standing can be tracked specifically by transcranial Doppler (TCD), which uses ultrasound to measure cerebral blood velocity (CBv) changes, most often in the middle cerebral artery (MCAv). These measurements reflect changes in CBF, provided that the artery diameter remains stable [22]. Until now, no studies have compared the oxygenation responses measured by long and short NIRS channels to postural BP changes.

The present study aimed to compare (cerebral) oxygenation obtained by NIRS with BP and MCAv during transitions from supine, sitting, or squatting to standing, stratified by age. Here, we recruited young and older adults not with the aim to investigate age-related changes in BP dynamics, but to elicit a wider range in postural BP responses. It was hypothesized that (a) BP would associate with O_2_Hb; (b) MCAv would associate with O_2_Hb, with the associations in (a) and (b) expected to be strongest in the early phase after standing, when BP changes are most pronounced and the short time window in which they occur limits compensation by cerebral autoregulation; and (c) associations between BP and short-channel O_2_Hb would be higher than for long-channel O_2_Hb, because cerebral autoregulation reduces the influence of BP on cerebral oxygenation but not on extracranial oxygenation, and that MCAv would show a stronger association with long-channel O_2_Hb than with short-channel O_2_Hb, reflecting changes in CBF.

## Methods

### Study design and participants

In a cross-sectional study at the geriatric department of the Radboudumc in Nijmegen, the Netherlands, between August 2021 and February 2022, 41 participants were included. To ensure a diversity of postural BP responses, both younger (18-35 years) and older adults (≥65 years) were included, with three times as many older adults, of whom at least 30% were recruited from the geriatric outpatient clinic. Participants were also recruited through flyers and advertisements at Radboud University. Exclusion criteria were being physically unable to perform orthostatic maneuvers or unable to understand written and oral instructions. Ethical approval was obtained from the medical ethics committee (CMO Arnhem-Nijmegen). All participants signed written informed consent. The study was performed in accordance with the declaration of Helsinki.

### Data collection

Information about age, height, weight, comorbidity, medication use (type and number of medications), alcohol use (units per week), smoking habits (yes/no), history of falls in the last year, and OH symptoms was obtained from all participants. Intake of caffeine, alcohol, medication, and food before the measurements was noted. Older participants (≥65 years) filled in The Older Persons and Informal Caregiver Survey-Short Form (TOPICS-SF) questionnaire on activities of daily living (ADL) and comorbidities [23], and completed the Montreal Cognitive Assessment (MoCA) as a cognitive screening tool [24]. All participants performed a maximum grip strength, grip work (sustained grip strength), and 5-times chair-stand test, as markers of global physical fitness.

All participants performed three supine-stand maneuvers and three sit-stand maneuvers. Participants were instructed to lie, sit, and stand still and not to talk during measurements. Instructions were given to perform the transitions as fast as possible. Supine and sitting resting periods lasted 5 min. After each postural transition, participants remained standing for 3 min. Three slower supine-stand maneuvers, where the transition was performed in approximately 10 s while the researcher counted from one to ten, and three squat-stand maneuvers, consisting of 1 min of squatting before standing, were performed additionally by participants between 18 and 35 years.

BP was measured continuously using volume-clamp photoplethysmography on the digital artery of the left middle finger (Finapres NOVA, Finapres Medical Systems, Enschede, the Netherlands) [25]. The hand was placed in a sling to prevent hydrostatic pressure artefacts.

(Cerebral) oxygenation was measured using two NIRS sensors bilaterally attached to the forehead approximately 2 cm above the eyebrows (PortaLite MkII, Artinis Medical Systems, Elst, the Netherlands), covered with a black bandana to prevent ambient light interference. Sensors consisted of three light-emitting diodes and two detectors, placed at inter-optode distances of 2.9, 3.5, and 4.1 cm for the three long channels and 0.70, 0.80, and 0.74 cm for the three short channels. The control unit was placed in a belt around the waist.

MCAv was measured using TCD through the temporal window (DWL Doppler Box, Compumedics Germany GmbH, Singen, Germany). TCD probes were fixed with a headband worn over the bandana. Heart rate was recorded using a three-lead electrocardiogram (ECG), and end-tidal carbon dioxide (etCO_2_) levels were measured using capnography, as an estimation for arterial partial pressure of CO_2_.

### Data acquisition and processing

(Cerebral) oxygenation signals were acquired in Oxysoft (version 3.4, Artinis Medical Systems, Elst, the Netherlands) at a sampling frequency of 100 Hz, while BP, MCAv, ECG, and etCO_2_ were obtained in Acqknowledge at 200 Hz (version 5.0, BioPac Systems Inc., Goleta, USA). Synchronization between both acquisition systems was achieved by analogue pulses (PortaSync MkII, Artinis Medical Systems, Elst, the Netherlands). Data were processed in MATLAB (2022a, MathWorks Inc., Natick, USA), using custom-written semi-automatic scripts [26]. Movement artefacts during supine-stand and sit-stand transitions were corrected in O_2_Hb and HHb signals during baseline and after 20 s of standing for each trial, using the movement artefact removal algorithm described by Scholkmann et al. [27, 28]. Between 2 s before standing up and 20 s after standing up (the period of the initial drop in O_2_Hb), movement artefacts could not be distinguished from postural oxygenation changes. Therefore, this part of the signal was not included in the movement artefact removal algorithm. Settings used were a threshold for artefact detection of three standard deviations, a moving window length for standard deviation calculation of one heartbeat (defined as sixty divided by the average heart rate during baseline or standing), and a 1-s local regression smoothing window for artefact correction. After this, the quality of all acquired signals was assessed visually, and signals not meeting the criteria were discarded. BP and MCAv signals were excluded when peaks and troughs were not distinguishable. ECG signals were excluded when QRS complexes were not visible. NIRS channels were excluded according to criteria described previously [29, 30]. These were an undistinguishable heartbeat during 10 s around the moment of standing up, flatlining, ambient light warnings, and an average tissue saturation index (TSI) fit factor (representing the agreement between different optodes) below 98%. Additionally, channels were excluded when movement artefacts were present during standing up, outside the correction window, or when the heartbeat amplitude was irregular (see Supplementary Table s1 and Fig. s2 for a full description of artefact types). Heart rate, systolic BP (SBP), diastolic BP (DBP), systolic MCAv (S-MCAv), diastolic MCAv (D-MCAv), and etCO_2_ were obtained over time by peak and trough detection. Mean MCAv, further referred to as MCAv, was calculated by two times D-MCAv and one time S-MCAv divided by three. tHb was calculated as the sum of O_2_Hb and HHb. All signals were resampled at 10 Hz and filtered using a 5-s moving average filter [31]. When bilateral MCAv and (cerebral) oxygenation signals were available and of sufficient quality, these were averaged. Otherwise, only the available signal was used for further analysis. For NIRS measurements, all available long channels were averaged, just like all available short channels.

### Signal phases and characteristics

**Fig. 1:**
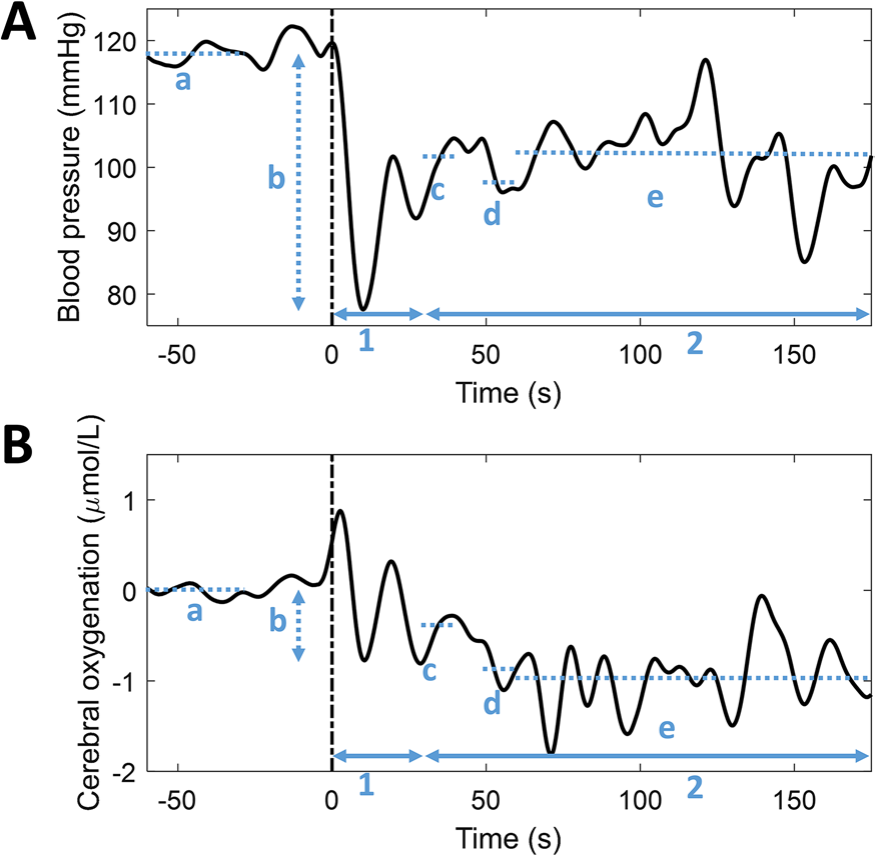
Example of **A** blood pressure response during standing and **B** oxygenation response during standing. Different phases (indicated by 1, 2) and characteristics (indicated by a, b, c, d, e) are shown in blue. 1, early recovery phase (0–30 s after standing up); 2, late recovery phase (30–175 s after standing up); and a, baseline, calculated as the average value between 60 and 30 s before standing up; b, initial drop within 30 s, calculated by minimum value minus baseline; c, early recovery, calculated as average value between 30 and 40 s after standing up; d, 1-min recovery, calculated as average value between 50 and 60 s after standing up; e, late recovery, calculated as average value between 60 and 175 s after standing up

Curves (see Fig. 1) were divided in an initial response phase (the first 30 s after standing up) and late response phase (from 30 s after standing up until the end of the measurement at 3 min of standing) for all signals (curve-based analysis).

Specific signal characteristics of the orthostatic courses of BP (systolic and diastolic), (cerebral) oxygenation (O_2_Hb and HHb in long and short channels), and MCAv were retrieved (visualized as b-e in Fig. 1) [15, 32]. These characteristics were the maximum drop amplitude, defined as the lowest value within 30 s after standing up, relative to the baseline value, and recovery at specified time points: the mean of 30-40 s (early recovery), the mean of 50–60 s (1-min recovery), and the mean of 60–175 s after standing up (late recovery), relative to the baseline value (characteristic-based analysis).

### Statistical analysis

Statistical analyses were performed in MATLAB (R2018a), IBM SPSS Statistics 27, and RStudio (2022.02.1). All continuous variables are presented as mean (standard deviation) for normally distributed data, or median (interquartile range) for otherwise distributed data. Categorical variables are presented as number (percentage). For all analyses, two-sided testing with an alpha level of 0.05 was used.

Curve-based Pearson correlation coefficients between BP and O_2_Hb were calculated for each postural change of each participant, during the initial and late response phases. Correlation coefficients were averaged over three repeats per type of postural change per participant, averaged over all participants, and classified as excellent (0.75–1), good (0.6–0.75), fair (0.4–0.6), or poor (0–0.4) [33, 34]. Signal characteristics (b–e in Fig. 1) were averaged over three repeats per participant if present and Pearson correlation coefficients between BP and O_2_Hb characteristics were calculated per type of postural change. The association between BP (both systolic and diastolic) and O_2_Hb (both long and short channels) considering both the supine-stand and sit-stand postural change was determined for the maximum drop value, the early recovery value, the 1-min recovery value, and the late recovery value (characteristics b–e in Fig. 1) in a multilevel model with O_2_Hb characteristics b-e as outcome variables. Fixed effects for the respective BP characteristics b-e and a random intercept for participants resulted in the best model fit, as evaluated using a likelihood ratio test.

Curve-based and characteristic-based associations between MCAv and O_2_Hb were determined similarly to the associations between O_2_Hb and BP. It was determined whether curve-based associations between BP or MCAv and long-channel O_2_Hb and between BP or MCAv and short-channel O_2_Hb were significantly different using paired *t*-tests. Differences between younger versus older adults in curve-based and characteristic-based associations were tested for significance using Mann-Whitney *U* tests.

## Results

### Baseline characteristics

Forty-one participants with a diversity of BP responses upon standing up were included of whom eleven were between 18 and 35 years old, and thirty were 65 years or older. Their baseline characteristics are presented in Table 1. Nine older participants had BP responses that fulfilled classic OH criteria during the supine-stand transitions. For one participant, fast supine-stand transitions were excluded due to technical issues. Supplementary Fig. s1 and s4 and Supplementary Table s2 further elaborate quality assessment, removal of artefacts, and number of transitions available for analyses. Of the 303 transitions, BP signals were present and of sufficient quality in almost all (301), TCD signals in two-third (213), long-channel NIRS in most (292), and short-channel NIRS in almost 80% (239).

**Table 1:** Baseline characteristics for younger and older participants, presented as mean (standard deviation (SD)) or median (interquartile range (IQR)) for continuous variables and number (percentage) for categorical variables

Characteristic	Older participants (*n* = 30)	Younger participants (*n* = 11)
Age (years), mean (SD)	74.2 (6.8)	24.6 (2.4)
Sex, female	11 (37)	7 (64)
BMI (kg/m^2^), mean (SD)	24.0 (3.1)	21.7 (1.8)
Smoking	1 (3.3)	0 (0)
Excessive alcohol use^a^	2 (6.7)	0 (0)
History of CVD	4 (13.3)	0 (0)
Diabetes mellitus	3 (10.0)	0 (0)
Orthostatic hypotension^b^	9 (30.0)	0 (0)
Initial orthostatic hypotension^c^	4 (13.3)	1 (9.1)
Symptoms of OH	16 (53.3)	10 (90.9)
During measurements	9 (30.0)	6 (54.6)
In daily life	15 (15.0)	10 (90.9)
Severe symptoms of OH^d^	4 (13.3)	1 (9.1)
During measurements	2 (6.7)	1 (9.1)
In daily life	2 (6.7)	0 (0)
MoCA, median (IQR)	26 (24-28)	NA
Medication use	23 (76.7)	2 (18.2)
Antihypertensive drug use	6 (20)	0 (0)
Statin use	3 (10)	0 (0)
Antidepressant use	2 (6.7)	0 (0)
Systolic BP (mmHg), mean (SD)^e^	141.2 (17.0)	126 (8.9)
Diastolic BP (mmHg), mean (SD)^e^	81.9 (10.2)	81 (9.0)
Maximum grip strength (kPa), mean (SD)	64.6 (18.0)	95.6 (18.1)
Grip work (kPa s), mean (SD)	2937 (1284)	3197 (1119)
Chair-stand test (s), mean (SD)	9.7 (2.7)	5.8 (0.9)

^a^Excessive alcohol use: more than 14 units per week for females and more than 21 units per week for males

^b^Measured with a continuous blood pressure device, and defined as systolic blood pressure (SBP) and/or diastolic blood pressure (DBP) dropping at least >20 or >10 mmHg once after 5-s moving average filtering in the period between 1 and 3 min after standing up [31]. A participant was classified as having orthostatic hypotension (OH) when the average of three supine-stand transitions met these criteria

^c^Defined as a SBP and/or DBP drop of at least 40 or 20 mmHg within the first 15 s after standing up [1]

^d^Defined as symptoms limiting standing or walking for a short (<1 min) or longer (>1 min) period of time

^e^Measured with an intermittent oscillometric device, as the average of two seated measurements

*MoCA*, Montreal cognitive assessment; *CVD*, cardiovascular disease; *BMI*, body mass index

### Average responses

**Fig. 2:**
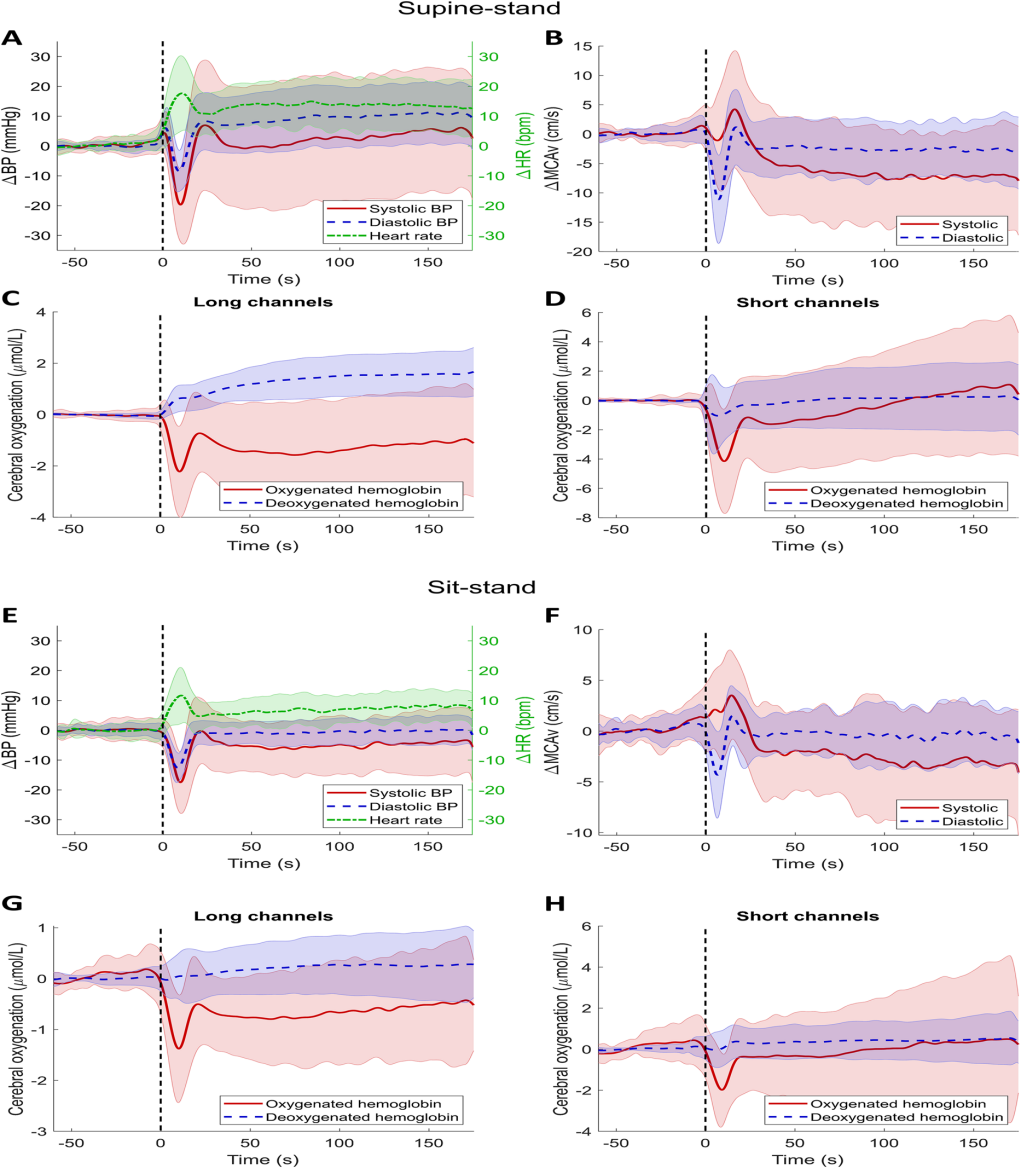
Responses of **A** + **E** blood pressure (BP), systolic in red (solid line), diastolic in blue (dashed line), and heart rate (HR) in green (dashed-dotted line); **B** + **F** cerebral blood velocity (MCAv), systolic in red (solid line), and diastolic in blue (dashed line); **C** + **G** cerebral oxygenation measured with long channels, oxygenated hemoglobin in red and deoxygenated hemoglobin in blue (dashed line); and **D** + **H** cerebral oxygenation measured with short channels, oxygenated hemoglobin in red and deoxygenated hemoglobin in blue (dashed line). All signals are shown from 1 min before standing up to 175 s after standing up, during a supine-stand transition (**A**–**D**) or sit-stand transition (**E**–**H**). These responses were averaged over all supine-stand or sit-stand transitions and all participants. Standing up is indicated by a vertical black dashed line. Shaded areas show standard deviations for all signals

Figure 2 shows the average BP, MCAv, and (cerebral) oxygenation responses for all participants, during all supine-stand and all sit-stand transitions. During a supine-stand postural change, the average initial SBP/DBP drop amplitude was −23.5/−10.4 (SD 14.1/7.2) mmHg, after which BP recovered to baseline values within 30 s from standing. S-MCAv and D-MCAv, which reflect systolic and diastolic CBF, showed different responses upon standing: whereas D-MCAv showed a drop (−12.6 (SD 8.3) cm/s), S-MCAv did not. This means that while, initially, systolic blood flow was maintained, diastolic flow was reduced, causing a reduction in total CBF during standing. In the early and late recovery phases, S-MCAv decreased and stabilized below baseline. This indicates that CBF upon standing does not fully return to supine values. O_2_Hb showed a clear drop upon standing, followed by recovery with an overshoot and stabilization to a level remaining below baseline (*p* < 0.001). The amplitude of the O_2_Hb drop was larger for short channels, on average −4.8 (SD 3.6) μmol/L, compared to −2.6 (SD 1.8) μmol/L for long channels (*p* < 0.001). When standing, long-channel HHb increased and stabilized above baseline (*p* = 0.001), while short-channel HHb showed a small decrease on average (*p* = 0.010). As tHb was calculated as the sum of O_2_Hb and HHb, and O_2_Hb showed more profound changes than HHb, the course of tHb was highly similar to the course of O_2_Hb (data not shown). In summary, there were reductions in long-channel O_2_Hb with concomitant increases in HHb in the later phase of standing.

For a sit-stand maneuver, the mean SBP/DBP drop amplitude upon standing was −20.8/−14.1 (SD 10.7/5.4) mmHg, after which BP recovered to baseline values within 20–30 s after standing up. Similar to the supine-stand test, S-MCAv did not show an initial drop upon standing, while D-MCAv did, on average −5.1 (SD 4.0) cm/s. O_2_Hb showed an initial drop in both long and short channels. Compared to the supine-stand test with an O_2_Hb drop of −2.6 (SD 1.8) μmol/L, this drop was smaller with −1.7 (SD 1.2) μmol/L in long channels (*p* = 0.008). Long-channel O_2_Hb did not return to the sitting baseline value (*p* < 0.001), in contrast to short-channel O_2_Hb. Long- and short-channel HHb both stabilized above baseline (*p* = 0.006 and *p* = 0.013 respectively). In summary, also after a sit-stand transition, TCD and NIRS signals indicate a small reduction in CBF upon standing after stabilization following the initial drop in BP and CBF.

The effects of aging on the responses to a postural change are shown in Supplementary Fig. s5 and s6. For both supine-stand and sit-stand transitions, younger adults show a larger drop in O_2_Hb immediately after standing compared to older adults, followed by a faster recovery. Younger adults also had a larger increase in heart rate upon standing.

For slow supine-stand and squat-stand in young adults, results are shown in Supplementary Fig. s3, showing that both short- and long-channel O_2_Hb followed BP changes and stabilized below baseline. The effects of postural changes on etCO_2_ are shown in Supplementary Fig. s4. During standing up from sitting or supine position, etCO_2_ levels decreased by 0.19–0.59%, both in the early and late recovery phase. This corresponds to a decrease of 1.4–4.2 mmHg [35]. During squatting, etCO_2_ levels increased, which continued in the early recovery phase, followed by a decrease in the late recovery phase.

### Associations between oxygenation and blood pressure

As shown in Table 2, curve-based Pearson correlation coefficients between BP and O_2_Hb were good (0.58–0.71) during the initial response but were poor (0.28–0.37) during the late response. All BP characteristics (maximum drop, early recovery, 1-min recovery, late recovery) showed poor associations with respective O_2_Hb characteristics (Table 3). These associations were all positive, indicating that a drop in BP led to a drop in O_2_Hb, for the supine-stand transition, and mostly positive for the sit-stand transition. Early and 1-min recovery BP values (both SBP and DBP) associated significantly with O_2_Hb values (Table 4). At maximum drop and late recovery, less consistent associations between BP and O_2_Hb characteristics were observed. They were only significant for maximum drop amplitude of DBP with long-channel O_2_Hb and late recovery of SBP with long-channel O_2_Hb. Sub-analyses (Supplementary Table s5) revealed higher Pearson correlation coefficients between BP and O_2_Hb for younger compared to older participants, which was significant for most associations.

**Table 2:**
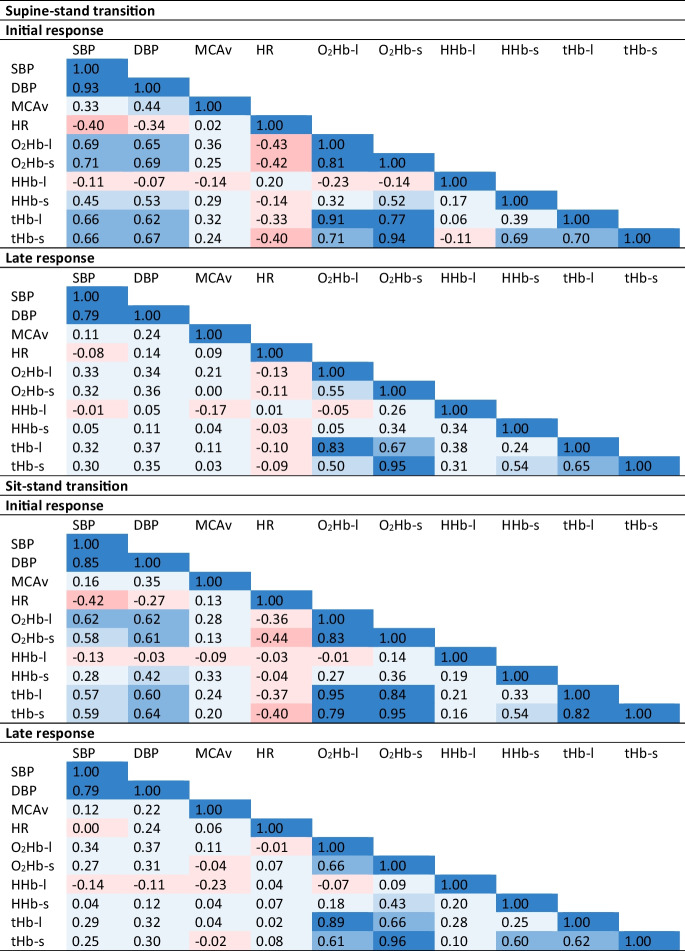
Heat map of average correlations for supine-stand and sit-stand transitions, during initial response (0–30 s after standing up) and late response (30–175 s after standing up)

*Significantly different (*p* < 0.05) between O_2_Hb-l and O_2_Hb-s. *SBP*, systolic blood pressure; *DBP*, diastolic blood pressure; *MCAv*, mean cerebral blood velocity in the middle cerebral artery; *O*_*2*_*Hb-l*, oxygenated hemoglobin measured with long channels; *O*_*2*_*Hb-s*, oxygenated hemoglobin measured with short channels; *HHb-l*, deoxygenated hemoglobin measured with long channels; *HHb-s*, deoxygenated hemoglobin measured with short channels

**Table 3:** Pearson correlation coefficients between characteristics derived from curves of systolic and diastolic blood pressure (SBP and DBP), cerebral blood velocity measured in the middle cerebral artery (MCAv), and oxygenated hemoglobin measured with long (O_2_Hb-l) and short (O_2_Hb-s) channels during a supine-stand transition and a sit-stand transition. Characteristics included in this table are maximum drop (minimum value within 30 s after standing up), early recovery (average value between 30 and 40 s after standing up with respect to baseline), 1-min recovery (average value between 50 and 60 s after standing up with respect to baseline), and late recovery (average value between 60 and 175 s after standing up with respect to baseline)

	Maximum drop amplitude	Early recovery	1-min recovery	Late recovery
Supine-stand				
SBP-O_2_Hb-l	0.131	0.226	0.372	0.319
SBP-O_2_Hb-s	0.131	0.168	0.219	0.114
DBP-O_2_Hb-l	0.279	0.178	0.281	0.253
DBP-O_2_Hb-s	0.259	0.218	0.250	0.148
MCAv-O_2_Hb-l	0.336	0.133	0.180	0.106
MCAv-O_2_Hb-s	0.114	−0.037	−0.128	−0.186
Sit-stand				
SBP-O_2_Hb-l	0.129	0.043	0.031	0.059
SBP-O_2_Hb-s	−0.011	0.157	−0.022	−0.060
DBP-O_2_Hb-l	0.365	−0.057	0.019	−0.056
DBP-O_2_Hb-s	0.214	0.181	0.156	0.119
MCAv-O_2_Hb-l	0.340	−0.273	−0.043	−0.066
MCAv-O_2_Hb-s	−0.014	−0.373	−0.264	−0.058

**Table 4:** Model results of a multilevel model with participants as random intercept and systolic or diastolic blood pressure (SBP or DBP) characteristics (maximum drop, early recovery, 1-min recovery, and late recovery) as fixed effects on (cerebral) oxygenation (O_2_Hb long and O_2_Hb short) characteristics (maximum drop, early recovery, 1-min recovery, late recovery). The three supine-stand and three sit-stand transitions are used, leading to six measurement values per participant

	Fixed effects		Random effects
	Estimate (95% CI)	*p*-value	SD intercept
O_2_Hb long ~ SBP (*n* = 221 (40))			
Maximum drop	0.011 (−0.006–0.027)	0.203	1.198
Early recovery (30–40 s)	0.019 (0.006–0.031)	0.003*	0.891
1-min recovery (50–60 s)	0.025 (0.011–0.040)	<0.001*	1.074
Late recovery (60–175 s)	0.017 (0.001–0.033)	0.033*	1.244
O_2_Hb short ~ SBP (*n* = 194 (40))			
Maximum drop	0.008 (−0.030–0.045)	0.692	2.214
Early recovery (30–40 s)	0.034 (0.005–0.062)	0.021*	1.833
1-min recovery (50–60 s)	0.026 (−0.005–0.056)	0.097	1.966
Late recovery (60–175 s)	0.012 (−0.018–0.042)	0.443	2.818
O_2_Hb long ~ DBP (*n* = 221 (40))			
Maximum drop	0.032 (0.002–0.063)	0.041*	1.142
Early recovery (30–40 s)	0.027 (0.002–0.051)	0.037*	0.881
1-min recovery (50–60 s)	0.028 (0.001–0.055)	0.045*	1.070
Late recovery (60–175 s)	0.018 (−0.010–0.047)	0.217	1.257
O_2_Hb short ~ DBP (*n* = 194 (40))			
Maximum drop	0.043 (−0.028–0.113)	0.240	2.133
Early recovery (30–40 s)	0.072 (0.013–0.131)	0.017*	1.803
1-min recovery (50–60 s)	0.063 (0.006–0.120)	0.032*	1.931
Late recovery (60–175 s)	0.023 (−0.034–0.079)	0.431	2.797

*Significant (*p* < 0.05) association of blood pressure characteristic with (cerebral) oxygenation characteristic

*SD*, standard deviation of the random intercept

### Associations between oxygenation and cerebral blood velocity

Table 2 shows that curve-based Pearson correlation coefficients between MCAv and O_2_Hb were lower than between BP and O_2_Hb. They were positive but poor during both the initial and late response. All MCAv characteristics showed positive poor associations with respective long-channel O_2_Hb characteristics, but much less so for short-channel O_2_Hb where only the maximum drop amplitude showed a positive association (Table 3). For sit-stand transitions, less consistent Pearson correlation coefficients between MCAv and O_2_Hb characteristics were found.

### Long- versus short-channel oxygenation

Curve- and characteristic-based associations (Tables 2 and 3) between BP and long-channel O_2_Hb and between BP and short-channel O_2_Hb were comparable. However, MCAv consistently showed stronger associations with long-channel O_2_Hb than with short-channel O_2_Hb, especially after 30 s of standing. Random effects (Table 4) showed higher between-subject variance in O_2_Hb characteristics for the short compared to the long channels.

## Discussion

### Main results

This cross-sectional study in 41 participants between 20 and 88 years old addressed the validity of measuring changes in (cerebral) oxygenation with NIRS to reflect the changes in BP and MCAv during postural changes. Good curve-based associations (0.6–0.8) between dynamic BP and O_2_Hb were found, albeit only for the initial response within the first 30 s after postural change. Early (30–40 s) and 1-min BP recovery were significantly associated with O_2_Hb, but there were no consistent associations for maximum drop amplitude and late recovery values. MCAv and O_2_Hb associated poorly, but MCAv associated consistently better with long-channel O_2_Hb than with short-channel O_2_Hb. Taken together, this indicates that in the early phase after standing (30 s), changes in O_2_Hb reflect changes in BP. Between 30 s and 1 min, O_2_Hb changes still indicate changes in BP, but based on the O_2_Hb signal alone, the magnitude of BP changes cannot be inferred. Long-channel NIRS signals correlate better with CBF (measured as changes in MCAv using TCD) than short-channel signals, indicating that short-channel NIRS measures extracranial (skin) tissue, but long-channel NIRS measures also cerebral tissue.

### (Cerebral) oxygenation compared to blood pressure responses to postural change

Long-channel O_2_Hb and HHb responses to postural change found in this study are consistent with previous studies: in young as well as older healthy adults, O_2_Hb and HHb did not return to baseline values, while BP did, even after 5 min of standing [15, 16, 36–38]. This is also reflected in the good curve-based Pearson correlation values, which showed high similarity between BP and O_2_Hb dynamics in the early recovery phase after standing up, confirming previous research [17], but this association disappeared in the late recovery phase. Supine-stand transitions showed slightly higher correlation coefficients between BP and O_2_Hb than sit-stand transitions. Standing up from a supine position leads to a more profound drop in O_2_Hb than standing up from a sitting position, probably because cerebral oxygenation is already lower in sitting compared to supine position [15, 16]. In this study, the larger O_2_Hb drop after a supine-stand transition was not accompanied by a larger BP drop, contrary to existing literature [39]. A lower cerebral oxygenation during sitting and standing could be explained by the differences in hydrostatic pressure gradient between the heart and brain in sitting or standing versus supine position, leading to a small but sustained reduction in cerebral perfusion pressure and thus cerebral oxygen delivery [21, 40, 41]. Because the cerebral metabolic demand and therefore oxygen consumption remain stable, this results in standing O_2_Hb values below and HHb values above baseline.

In the late recovery phase, cerebral autoregulation has had time to react to BP changes, resulting in a new equilibrium wherein there is no longer an influence of BP on CBF. O_2_Hb and BP were significantly associated for early and 1-min recovery values, but did not strongly associate characteristic-based, and thus between participants, suggesting that subject-specific characteristics may be an important source of variance. This was supported by the fact that a random, participant-dependent, intercept improved the multilevel model fit, indicating that an average BP drop is accompanied by a subject-specific oxygenation drop. Age may play a role, as younger participants had a better curve-based association between O_2_Hb and BP in the initial phase than older participants.

### (Cerebral) oxygenation measured with long channels and short channels

O_2_Hb and MCAv both dropped upon standing. Compared to associations between BP and O_2_Hb, MCAv and O_2_Hb associated only poorly. This indicates that not only cerebral effects have been reflected in O_2_Hb, but also extracranial blood flow. However, both curve-based and characteristic-based associations were consistently higher between MCAv and long-channel O_2_Hb than between MCAv and short-channel O_2_Hb, while BP associated similarly well with both long- and short-channel O_2_Hb. This corresponds to our hypothesis and underpins the potential value of discerning between long and short channels, in which long-channel signals are expected to better reflect the effects of cerebral autoregulation, and thus associate better with MCAv, than short-channel signals which are thought to reflect superficial, extracerebral blood flow changes. In further support of this, in the later phase (>30 s) after postural change, when compensation by cerebral autoregulation has taken place, the difference between long and short channels became more pronounced.

Although the combination of long and short channels is promising, short channels showed a few disadvantages in our study. Compared to long-channel oxygenation, short-channel O_2_Hb and HHb responses were more heterogeneous, both during a supine-stand and a sit-stand transition, and less robust than long-channel measurements. This was expressed in the number of repetitions that had to be discarded due to bad signal quality, like movement artefacts, and irregular heartbeat amplitudes that were not present in long channels.

### Importance of cerebral oxygenation measurements

Our results indicate that there is no linear relation between O_2_Hb and BP, and that linear methods as used in this study are not sufficient for an absolute BP estimation using oxygenation signals, as characteristic-based associations between O_2_Hb and BP were all poor. It is however questionable whether absolute BP estimation should be targeted, because orthostatic intolerance is not only determined by absolute BP values. Orthostatic intolerance symptoms (light-headedness, dizziness, blurred vision) as well as negative health outcomes related to OH such as falls and fractures all originate from cerebral hypoperfusion [4, 21, 42, 43]. During postural changes, the reduction in CBF is not solely dependent on BP, but also on the efficiency of cerebral autoregulation, on the magnitude of changes in etCO_2_, and on the hydrostatic gradient between the heart and brain [21, 44–46]. Measuring cerebral oxygenation, which reflects cerebral perfusion, may therefore have a more direct association with symptoms and outcomes than measuring BP. Previous research by the TILDA group, linking low cerebral oxygenation to depression, suggests this mediating role [47, 48]. However, it is good to realize that cerebral hypoperfusion is not always symptomatic. Orthostatic cerebral oxygenation in the same cohort was not associated with orthostatic intolerance symptoms [49]. Orthostatic intolerance symptoms were defined as the presence of any symptoms of dizziness, light-headedness, or unsteadiness, in which symptom severity was not considered. In the present study, more than 50% of young participants reported symptoms, although it is unlikely that they suffer from orthostatic intolerance impairing daily life or mediating negative health outcomes. Moreover, recent research suggests that patients who have OH are often not recognizing accompanying symptoms themselves, making them at risk of falls [50]. Therefore, cerebral oxygenation in relation to orthostatic intolerance is a topic for further research, especially in the home setting where falls occur. Eventually, this may aid in reconsidering the OH definition, from BP-based to cerebral oxygenation-based.

### Strengths and limitations

Strengths of this study were the multimodal measurements, use of different postural changes, each repeated three times, and a heterogeneous cohort with a broad age range, the latter two leading to a large variety of BP responses. This study had a few limitations. First, since the PortaLite MkII was still being improved during this study, some channels had to be excluded due to noise which was present in one sensor. Second, different repetitions were excluded due to artefacts, which were classified arbitrarily. However, to ensure consistency, classification rules were set, as described in the Supplementary Information, and classification was performed by two researchers independently. Third, the TCD headband was placed over the NIRS sensors, because of limited space on the forehead. This complicated MCAv recordings, and therefore led to TCD measurements being unavailable in one-third of all subjects. This however reflects reality, as TCD is less often measurable in older adults, and supports the use of NIRS for cerebral perfusion monitoring, as sensor attachment is less complex and time-consuming than for TCD. Fourth, we did not specifically target to include participants with OH, and therefore, only a limited number with OH were included, especially a small number of participants with orthostatic intolerance symptoms. Therefore, assessment of the relation between O_2_Hb after postural change and orthostatic intolerance was not possible. Finally, future work is needed to investigate the effects of dynamic cerebral autoregulation on the relationship between BP and CBF and cerebral oxygenation during postural changes. Such studies should expand beyond linear statistics and investigate the non-linear dynamics of these signals, both in the time and frequency domain [51].

### Conclusion

In conclusion, our results indicate that O_2_Hb measured with NIRS can capture postural BP dynamics. The larger the change in O_2_Hb, for example in the initial response phase or a supine-stand instead of sit-stand transition, the better O_2_Hb is reflecting BP changes. Small changes, as present in the late response phase after postural change, were not directly transferred from BP to oxygenation. Long-channel O_2_Hb probably captured part of the cerebral response, contrary to short-channel O_2_Hb, and is therefore promising for measuring orthostatic intolerance, as these symptoms are not always directly related to OH. As such, future research should focus on measuring (cerebral) oxygenation in a daily life setting, to investigate NIRS as an ambulant monitoring tool for OH and orthostatic intolerance.

### Supplementary information

ESM 1: Fig. s1 Flowchart of available participants, maneuvers, repetitions and signals, that were included in the analyses (available data fulfilling quality criteria). Fig. s2 Examples of different artefacts that were seen in the near-infrared spectroscopy (NIRS) data. Table s1 Description of artefacts that were present in the data. Table s2 Number of excluded channels due to artefacts per type of maneuver and type of near-infrared spectroscopy (NIRS) channel (short or long), specified by type of artefact. Fig. s3 Responses of blood pressure, heart rate, cerebral blood velocity, cerebral oxygenation measured with long channelsand cerebral oxygenation measured with short channels, averaged over all supine-stand or squat-stand transitionsand all young participants. Table s3 Heatmaps of average correlations during a slow supine-stand transition. Table s4 Heatmaps of average correlations during a squat-stand transition. Fig. s4 Responses of end-tidal carbon dioxide (etCO2) during a fast supine-stand, sit-stand, slow supine-stand and squat-stand transition. Table s5 Heatmaps of average correlations during a fast supine-stand transition, showing sub-analyses for younger (18-35 years) and older (>65 years) adults. Fig. s5 Supine-stand responses in older (>65 years) and younger (18-35 years) participants Fig. s6 Sit-stand responses in older (>65 years) and younger (18-35 years) participants.
