# Correction to: Capturing postural blood pressure dynamics with near‑infrared spectroscopy‑measured cerebral oxygenation

**DOI:** 10.1007/s11357-023-00837-y

**Published:** 2023-05-31

**Authors:** Marjolein Klop, Rianne A. A. de Heus, Andrea B. Maier, Anne van Alphen, Marianne J. Floor‑Westerdijk, Mathijs Bronkhorst, René J. F. Melis, Carel G. M. Meskers, Jurgen A. H. R. Claassen, Richard J. A. van Wezel

**Affiliations:** 1 Department of Biophysics, Donders Institute for Brain, Cognition and Behaviour, Radboud University, Nijmegen, the Netherlands; 2 Department of Geriatric Medicine, Radboud University Medical Center, Nijmegen, the Netherlands; 3 Department of Primary and Community Care, Radboud University Medical Center, Nijmegen, the Netherlands; 4 Department of Human Movement Sciences, @AgeAmsterdam, Amsterdam Movement Sciences, Vrije Universiteit Amsterdam, Amsterdam, the Netherlands; 5 Healthy Longevity Translational Research Program, Yong Loo Lin School of Medicine, National University of Singapore, Singapore, Singapore; 6 Centre for Healthy Longevity, @AgeSingapore, National University Health System, Singapore, Singapore; 7 Artinis Medical Systems, Elst, the Netherlands; 8 Department of Rehabilitation Medicine, Amsterdam Movement Sciences, Amsterdam University Medical Center, Amsterdam, the Netherlands; 9 Department of Cardiovascular Sciences, University of Leicester, Leicester, UK; 10 Department of Biomedical Signals and Systems, Technical Medical Centre, University of Twente, Enschede, the Netherlands

**Correction to: GeroScience**

https://doi.org/10.1007/s11357-023-00791-9

In the article version originally published online, Table 2 was switched in the publication process. The correct version of Table 2 is shown below.

**Table 2:**
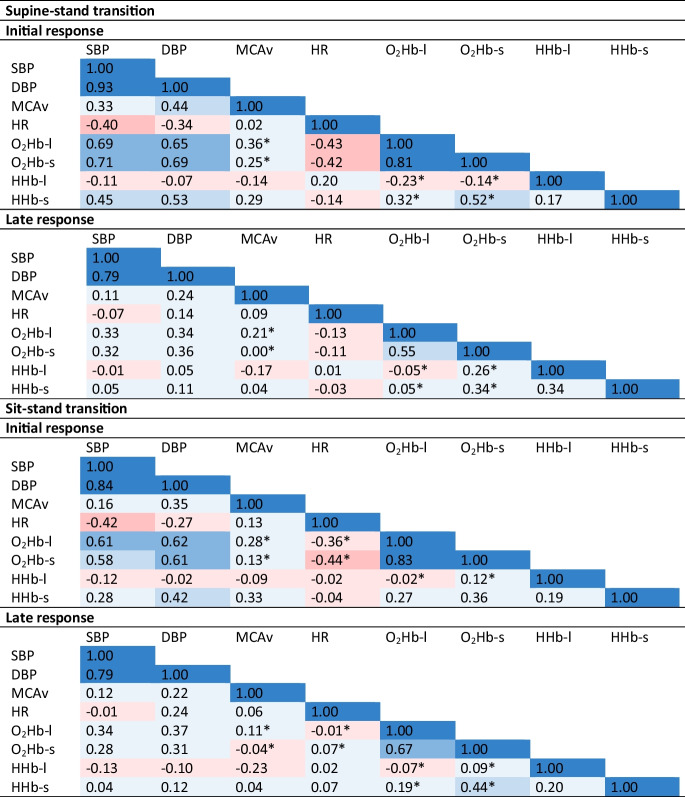
Heat map of average correlations for supine-stand and sit-stand transitions, during initial response (0-30 seconds after standing up) and late response (30-175 seconds after standing up)

* Significantly different (*p*<0.05) between O_2_Hb-l and O_2_Hb-s. *SBP* Systolic blood pressure; *DBP* Diastolic blood pressure; *MCAv* Mean cerebral blood velocity in the middle cerebral artery; *O*_*2*_*Hb-l* Oxygenated hemoglobin measured with long channels; *O*_*2*_*Hb-s* Oxygenated hemoglobin measured with short channels; *HHb-l* Deoxygenated hemoglobin measured with long channels; *HHb-s* Deoxygenated hemoglobin measured with short channels

