# Supplementary material for: Capturing postural blood pressure dynamics with near-infrared spectroscopy-measured cerebral oxygenation
Source: GeroScience. 2023 Apr 12;45(4):2643–57. doi: 10.1007/s11357-023-00791-9 (PMC10651596; doi:10.1007/s11357-023-00791-9)
Supplement: Supplementary file 1 — Fig. s1 Flowchart of available participants, maneuvers, repetitions and signals, that were included in the analyses (available data fulfilling quality criteria). Fig. s2 Examples of different artefacts that were seen in the near-infrared spectroscopy (NIRS) data. Table s1 Description of artefacts that were present in the data. Table s2 Number of excluded channels due to artefacts per type of maneuver and type of near-infrared spectroscopy (NIRS) channel (short or long), specified by type of artefact. Fig. s3 Responses of blood pressure, heart rate, cerebral blood velocity, cerebral oxygenation measured with long channelsand cerebral oxygenation measured with short channels, averaged over all supine-stand or squat-stand transitionsand all young participants. Table s3 Heatmaps of average correlations during a slow supine-stand transition. Table s4 Heatmaps of average correlations during a squat-stand transition. Fig. s4 Responses of end-tidal carbon dioxide (etCO2) during a fast supine-stand, sit-stand, slow supine-stand and squat-stand transition. Table s5 Heatmaps of average correlations during a fast supine-stand transition, showing sub-analyses for younger (18-35 years) and older (>65 years) adults. Fig. s5 Supine-stand responses in older (>65 years) and younger (18-35 years) participants Fig. s6 Sit-stand responses in older (>65 years) and younger (18-35 years) participants. [file 11357_2023_791_MOESM1_ESM.docx]

**Supplementary material**

**Capturing postural blood pressure dynamics with near-infrared spectroscopy-measured cerebral oxygenation**

**Marjolein Klop*, Rianne A.A. de Heus, Andrea B. Maier, Anne van Alphen, Marianne J. Floor-Westerdijk, Mathijs Bronkhorst, René J.F. Melis, Carel G.M. Meskers, Jurgen A.H.R. Claassen, Richard J.A. van Wezel**

* Corresponding author:

Marjolein Klop

Department of Biophysics, Donders Institute for Brain, Cognition and Behaviour, Radboud University, Nijmegen, The Netherlands

marjolein.klop@donders.ru.nl

***Included signals***


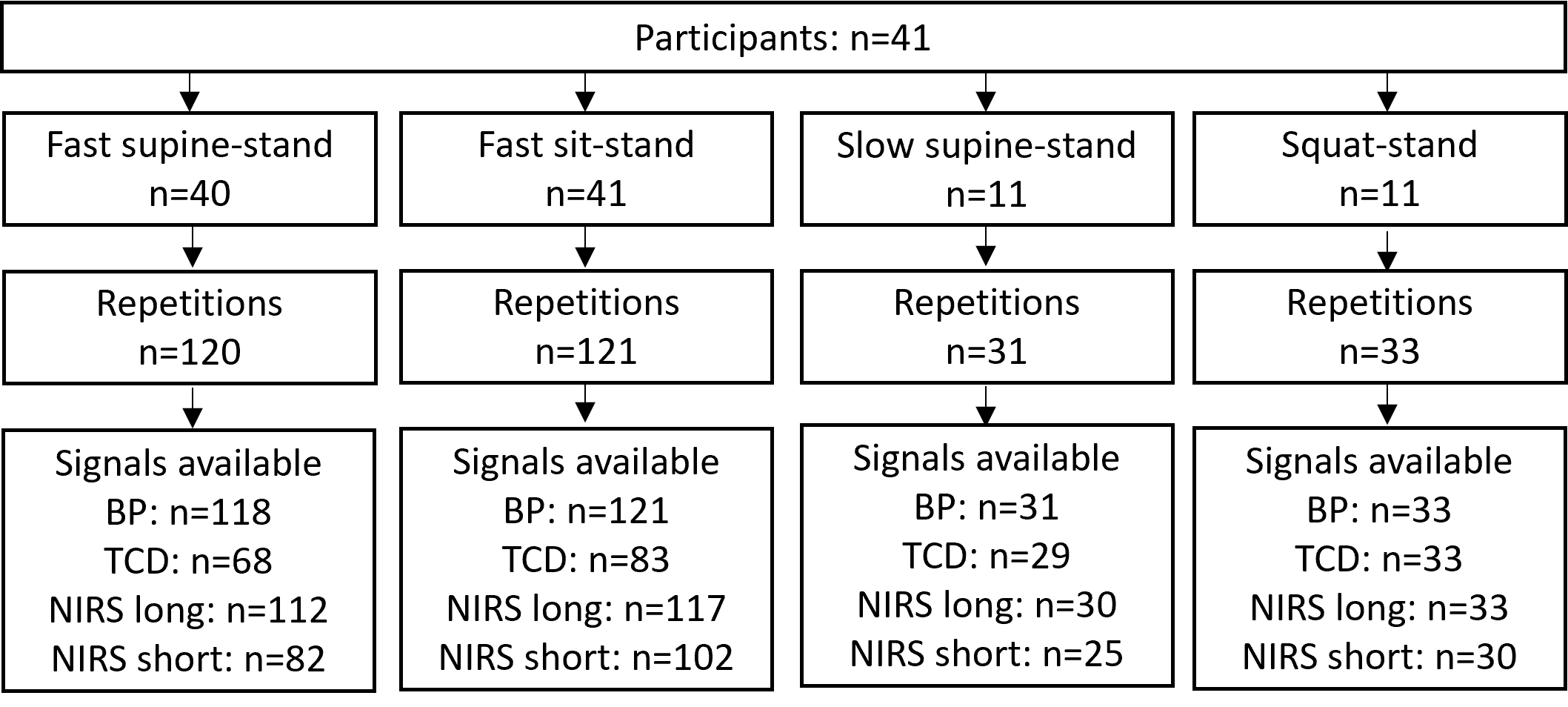


**Fig. s1 Flowchart of available participants, maneuvers, repetitions and signals, that were included in the analyses (available data fulfilling quality criteria). BP: blood pressure, TCD: transcranial Doppler, NIRS: near-infrared spectroscopy. NIRS long: oxygenation measured with long (3-4 cm) channels, NIRS short: oxygenation measured with short (<1 cm) channels.**

***NIRS artefacts***

**Fig. s2 Examples of different artefacts that were seen in the near-infrared spectroscopy (NIRS) data. The graphs represent oxygenated hemoglobin signals, with A loss of signal during a time window of 30 seconds in a long NIRS channel, B noise during a time window of 30 seconds in a long NIRS channel, C irregular heart beat amplitude over four minutes in a short NIRS channel, D magnified segment from the irregular heart beat signal during 10 seconds, E movement artefact during a time window of 60 seconds in a long NIRS channel, F corrected movement artefact by the movement artefact removal algorithm (MARA) [1] with the corrected oxygenated hemoglobin shown in red, and the uncorrected in black, during a time window of 100 seconds.**

**Table s1 Description of artefacts that were present in the data. Artefacts that were identified consisted of loss of signal, noise, irregular amplitude and movement artefacts.**

| **Artefact name** | **Description** |
| --- | --- |
| **Loss of signal** | **Sudden flat line or high amplitude ‘blocks’ in the signal, caused by for example loss of Bluetooth connection between the sensor control unit and the acquisition software on a laptop or problems with the physical connection between sensor and control unit.** |
| **Noise** | **Signal with a high frequent component, in which heartbeats (saw-tooth waveform caused by heartbeats) are no longer visible.** |
| **Irregular amplitude** | **Amplitude of the heartbeat that is visible in the NIRS signal differs much over time. Moreover, the shape of the heartbeat signal is different (more spiky) than usually. This only happened in short NIRS channels.** |
| **Movement artefact** | **Sudden peak in the NIRS signal, at least 3 times larger than the standard deviation of the signal, which is sometimes accompanied by a baseline shift of the NIRS signal. During baseline and during standing (after stabilization of the blood pressure), this could be fixed in some cases by using a movement artefact removal algorithm [1].** |

**Table s2 Number of excluded channels due to artefacts per type of maneuver and type of near-infrared spectroscopy (NIRS) channel (short or long), specified by type of artefact. The total number (n) indicated per maneuver type is the number of participants times six (three repetitions of each transition, and two sensors (left and right) placed on the forehead). Results are noted as number (percentage of total number of channels).**

| **Maneuver type** | **Fast supine-stand (n=240)** | | **Fast sit-stand (n=246)** | | **Slow supine-stand (n=66)** | | **Squat-stand (n=66)** | |
| --- | --- | --- | --- | --- | --- | --- | --- | --- |
| **Channel** | **Long** | **Short** | **Long** | **Short** | **Long** | **Short** | **Long** | **Short** |
| **No NIRS signal available** | **9 (4)** | **9 (4)** | **7 (3)** | **10 (4)** | **4 (6)** | **4 (6)** | **0** | **0** |
| **Loss of signal** | **14 (6)** | **8 (3)** | **16 (7)** | **6 (2)** | **0 (0)** | **0 (0)** | **0** | **0** |
| **Noise** | **11 (5)** | **20 (8)** | **7 (3)** | **14 (6)** | **8 (12)** | **3 (5)** | **6 (9)** | **0** |
| **Irregular amplitude** | **0 (0)** | **45 (19)** | **0 (0)** | **17 (7)** | **0** | **11 (17)** | **0** | **0** |
| **Movement artefact** | **24 (10)** | **33 (14)** | **7 (3)** | **17 (7)** | **0** | **1 (2)** | **1 (2)** | **7 (11)** |
| **Movement artefacts solved by MARA** | **3 (1)** | **9 (4)** | **7 (3)** | **9 (4)** | **-** | **-** | **-** | **-** |

**MARA: movement artefact removal algorithm.**

***Slow supine-stand and squat-stand***

**Fig. s3** **Responses of A+E blood pressure (BP), systolic in red (solid line), diastolic in blue (dashed line), and heart rate (HR) in green (dashed-dotted line), B+F cerebral blood velocity (MCAv), systolic in red (solid line), diastolic in blue (dashed line), C+G cerebral oxygenation measured with long channels, oxygenated hemoglobin in red (solid line) and deoxygenated hemoglobin in blue (dashed line) and D+H) cerebral oxygenation measured with short channels, oxygenated hemoglobin in red (solid line) and deoxygenated hemoglobin in blue (dashed line). All signals are shown from 60 (slow supine-stand) or 55 seconds (squat-stand) before standing up to 175 seconds after standing up, during a slow supine-stand challenge. These responses were averaged over all supine-stand (A-D) or squat-stand transitions (E-H) and all young participants. Standing up is indicated by a black dashed line. Shaded areas show standard deviations for all signals.**

**Table s3 Heatmaps of average correlations during a slow supine-stand transition, during initial response (0-30 seconds after standing up) and late response (30-175 seconds after standing up).**

| **Initial response** |
| --- |
| \|  \| SBP \| DBP \| MCAv \| HR \| O_2_Hb-l \| O_2_Hb-s \| HHb-l \| HHb-s \| \| --- \| --- \| --- \| --- \| --- \| --- \| --- \| --- \| --- \| \| SBP \| 1.00 \|  \|  \|  \|  \|  \|  \|  \| \| DBP \| 0.95 \| 1.00 \|  \|  \|  \|  \|  \|  \| \| MCAv \| 0.35 \| 0.48 \| 1.00 \|  \|  \|  \|  \|  \| \| HR \| -0.69 \| -0.63 \| 0.02 \| 1.00 \|  \|  \|  \|  \| \| O_2_Hb-l \| 0.66 \| 0.67 \| 0.37* \| -0.65* \| 1.00 \|  \|  \|  \| \| O_2_Hb-s \| 0.75 \| 0.75 \| 0.22* \| -0.82* \| 0.83 \| 1.00 \|  \|  \| \| HHb-l \| 0.22 \| 0.15 \| -0.38 \| -0.06 \| -0.23* \| 0.00* \| 1.00 \|  \| \| HHb-s \| 0.69 \| 0.71 \| 0.09 \| -0.55 \| 0.41* \| 0.65* \| 0.40 \| 1.00 \| |
| **Late response** |
| \|  \| SBP \| DBP \| MCAv \| HR \| O_2_Hb-l \| O_2_Hb-s \| HHb-l \| HHb-s \| \| --- \| --- \| --- \| --- \| --- \| --- \| --- \| --- \| --- \| \| SBP \| 1.00 \|  \|  \|  \|  \|  \|  \|  \| \| DBP \| 0.45 \| 1.00 \|  \|  \|  \|  \|  \|  \| \| MCAv \| 0.09 \| 0.12 \| 1.00 \|  \|  \|  \|  \|  \| \| HR \| -0.41 \| 0.15 \| -0.04 \| 1.00 \|  \|  \|  \|  \| \| O_2_Hb-l \| 0.41 \| 0.15* \| 0.29* \| -0.37* \| 1.00 \|  \|  \|  \| \| O_2_Hb-s \| 0.34 \| 0.40* \| -0.05* \| -0.15* \| 0.65 \| 1.00 \|  \|  \| \| HHb-l \| -0.25 \| 0.20 \| -0.44 \| 0.28 \| -0.38* \| 0.23* \| 1.00 \|  \| \| HHb-s \| -0.08 \| 0.31 \| -0.23 \| 0.15 \| -0.01* \| 0.36* \| 0.67 \| 1.00 \| |
| **SBP: Systolic blood pressure, DBP: diastolic blood pressure, MCAv: mean blood flow velocity in middle cerebral artery, O_2_Hb-l: oxygenated hemoglobin measured with long channels, O_2_Hb-s: oxygenated hemoglobin measured with short channels, HHb-l: deoxygenated hemoglobin measured with long channels, HHb-s: deoxygenated hemoglobin measured with short channels.** |

**Table s4 Heatmaps of average correlations during a squat-stand transition, during initial response (0-30 seconds after standing up) and late response (30-175 seconds after standing up).**

| **Initial response** |
| --- |
| \|  \| SBP \| DBP \| MCAv \| HR \| O_2_Hb-l \| O_2_Hb-s \| HHb-l \| HHb-s \| \| --- \| --- \| --- \| --- \| --- \| --- \| --- \| --- \| --- \| \| SBP \| 1.00 \|  \|  \|  \|  \|  \|  \|  \| \| DBP \| 0.77 \| 1.00 \|  \|  \|  \|  \|  \|  \| \| MCAv \| 0.01 \| 0.14 \| 1.00 \|  \|  \|  \|  \|  \| \| HR \| -0.27 \| -0.38 \| -0.37 \| 1.00 \|  \|  \|  \|  \| \| O_2_Hb-l \| 0.63 \| 0.65 \| -0.26 \| -0.04 \| 1.00 \|  \|  \|  \| \| O_2_Hb-s \| 0.66 \| 0.67 \| -0.25 \| -0.06 \| 0.91 \| 1.00 \|  \|  \| \| HHb-l \| 0.15 \| 0.33 \| 0.03 \| -0.35 \| -0.02* \| 0.12* \| 1.00 \|  \| \| HHb-s \| 0.35 \| 0.55 \| 0.54 \| -0.71 \| 0.11 \| 0.21 \| 0.42 \| 1.00 \| |
| **Late response** |
| \|  \| SBP \| DBP \| MCAv \| HR \| O_2_Hb-l \| O_2_Hb-s \| HHb-l \| HHb-s \| \| --- \| --- \| --- \| --- \| --- \| --- \| --- \| --- \| --- \| \| SBP \| 1.00 \|  \|  \|  \|  \|  \|  \|  \| \| DBP \| 0.34 \| 1.00 \|  \|  \|  \|  \|  \|  \| \| MCAv \| 0.41 \| -0.11 \| 1.00 \|  \|  \|  \|  \|  \| \| HR \| -0.13 \| -0.15 \| 0.33 \| 1.00 \|  \|  \|  \|  \| \| O_2_Hb-l \| 0.35 \| 0.20 \| 0.24* \| -0.14 \| 1.00 \|  \|  \|  \| \| O_2_Hb-s \| 0.08 \| 0.33 \| -0.22* \| -0.34 \| 0.47 \| 1.00 \|  \|  \| \| HHb-l \| -0.21 \| 0.16 \| -0.51 \| -0.26 \| -0.16* \| 0.28* \| 1.00 \|  \| \| HHb-s \| 0.08 \| 0.19 \| 0.06 \| -0.18 \| 0.31 \| 0.33 \| 0.31 \| 1.00 \| |
| **SBP: Systolic blood pressure, DBP: diastolic blood pressure, MCAv: mean blood flow velocity in middle cerebral artery, O_2_Hb-l: oxygenated hemoglobin measured with long channels, O_2_Hb-s: oxygenated hemoglobin measured with short channels, HHb-l: deoxygenated hemoglobin measured with long channels, HHb-s: deoxygenated hemoglobin measured with short channels.** |

***End-tidal CO_2_***


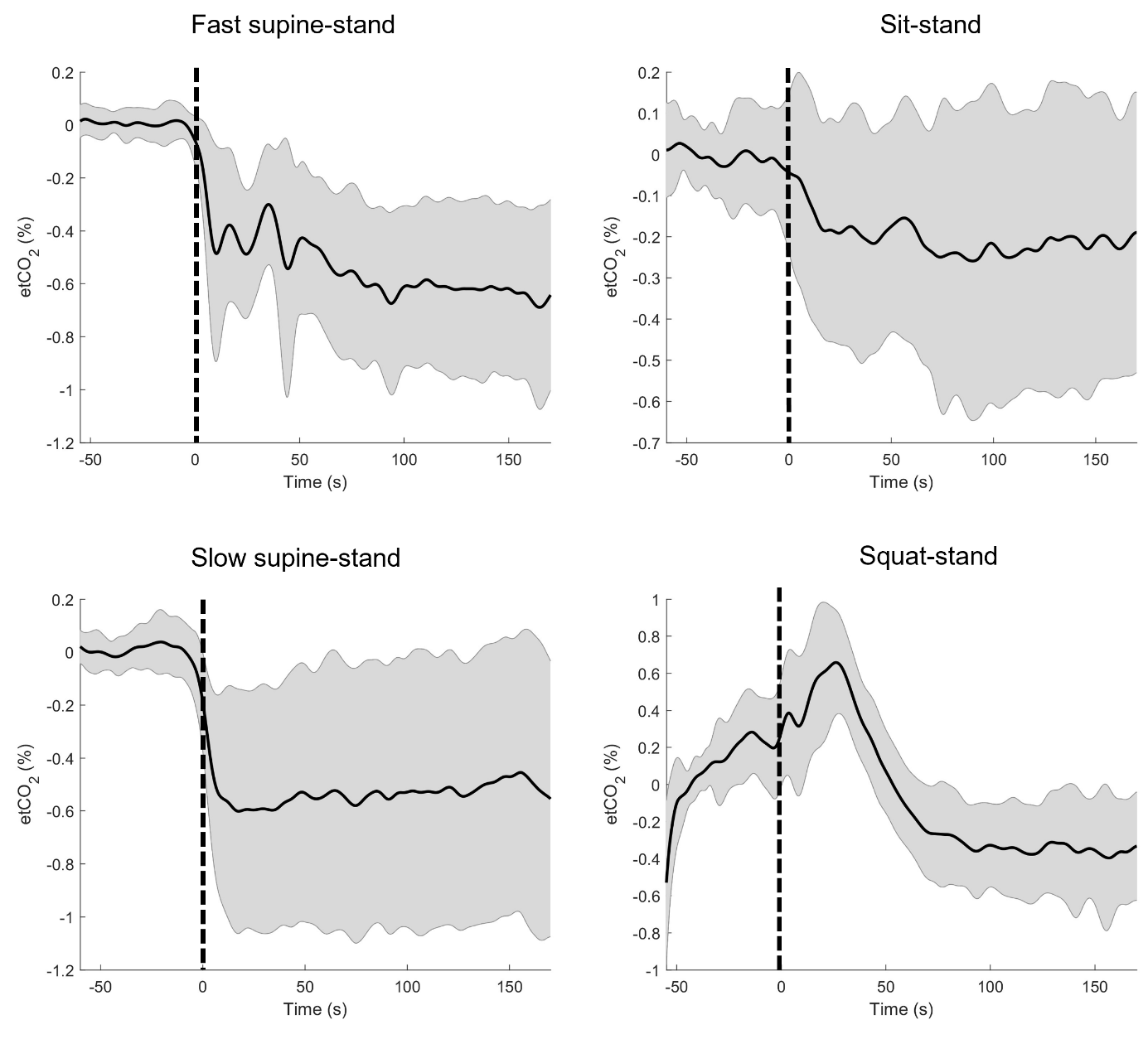


**Fig. s4** **Responses of end-tidal carbon dioxide (etCO_2_) during a fast supine-stand, sit-stand, slow supine-stand and squat-stand transition. All signals are shown from 60 seconds (or 55 seconds in case of squat-stand) before standing up to 170 seconds after standing up. These responses were averaged over all transitions and all participants. Standing up is indicated by a dashed line. Shaded areas show standard**

**Sub-analysis younger versus older participants**

**Table s5 Heatmaps of average correlations during a fast supine-stand transition, during baseline (1 minute before standing up), initial response (0-30 seconds after standing up) and late response (30-175 seconds after standing up). These heatmaps show sub-analyses for young (18-35 years) and older (>65 years) adults.**

| **Supine-stand** | |  |  |  |
| --- | --- | --- | --- | --- |
| **Younger (18-35 years) adults (n=10)** | |  |  |  |
| **Initial response** | | | | |
| \|  \| SBP \| DBP \| MCAv \| HR \| O_2_Hb-l \| O_2_Hb-s \| HHb-l \| HHb-s \| \| --- \| --- \| --- \| --- \| --- \| --- \| --- \| --- \| --- \| \| SBP \| 1.00 \|  \|  \|  \|  \|  \|  \|  \| \| DBP \| 0.95 \| 1.00 \|  \|  \|  \|  \|  \|  \| \| MCAv \| 0.41 \| 0.51 \| 1.00 \|  \|  \|  \|  \|  \| \| HR \| -0.77* \| -0.68* \| -0.06 \| 1.00 \|  \|  \|  \|  \| \| O_2_Hb-l \| 0.84* \| 0.79* \| 0.36 \| -0.75* \| 1.00 \|  \|  \|  \| \| O_2_Hb-s \| 0.80 \| 0.73 \| 0.22 \| -0.82* \| 0.89 \| 1.00 \|  \|  \| \| HHb-l \| 0.13 \| 0.13 \| -0.12 \| -0.11* \| -0.08 \| 0.07 \| 1.00 \|  \| \| HHb-s \| 0.83* \| 0.83* \| 0.37 \| -0.68* \| 0.70* \| 0.77 \| 0.18 \| 1.00 \| | | | | |
| **Late response** | | | | |
| \|  \| SBP \| DBP \| MCAv \| HR \| O_2_Hb-l \| O_2_Hb-s \| HHb-l \| HHb-s \| \| --- \| --- \| --- \| --- \| --- \| --- \| --- \| --- \| --- \| \| SBP \| 1.00 \|  \|  \|  \|  \|  \|  \|  \| \| DBP \| 0.61* \| 1.00 \|  \|  \|  \|  \|  \|  \| \| MCAv \| 0.10 \| 0.18 \| 1.00 \|  \|  \|  \|  \|  \| \| HR \| -0.41* \| -0.02* \| 0.12 \| 1.00 \|  \|  \|  \|  \| \| O_2_Hb-l \| 0.34 \| 0.27 \| 0.26 \| -0.18 \| 1.00 \|  \|  \|  \| \| O_2_Hb-s \| 0.21 \| 0.40 \| -0.02 \| 0.01 \| 0.40 \| 1.00 \|  \|  \| \| HHb-l \| -0.14 \| 0.13 \| -0.26 \| 0.19 \| -0.22 \| 0.37 \| 1.00 \|  \| \| HHb-s \| -0.11 \| 0.17 \| 0.00 \| 0.18* \| -0.01 \| 0.44 \| 0.44 \| 1.00 \| | | | | |
| **Older (>65 years) adults (n=30)** |  |  |  |  |
| **Initial response** | | | | |
| \|  \| SBP \| DBP \| MCAv \| HR \| O_2_Hb-l \| O_2_Hb-s \| HHb-l \| HHb-s \| \| --- \| --- \| --- \| --- \| --- \| --- \| --- \| --- \| --- \| \| SBP \| 1.00 \|  \|  \|  \|  \|  \|  \|  \| \| DBP \| 0.92 \| 1.00 \|  \|  \|  \|  \|  \|  \| \| MCAv \| 0.29 \| 0.40 \| 1.00 \|  \|  \|  \|  \|  \| \| HR \| -0.27* \| -0.22* \| 0.06 \| 1.00 \|  \|  \|  \|  \| \| O_2_Hb-l \| 0.64* \| 0.60* \| 0.37 \| -0.32* \| 1.00 \|  \|  \|  \| \| O_2_Hb-s \| 0.68 \| 0.67 \| 0.28 \| -0.26* \| 0.77 \| 1.00 \|  \|  \| \| HHb-l \| -0.19 \| -0.14 \| -0.15 \| 0.30* \| -0.28 \| -0.23 \| 1.00 \|  \| \| HHb-s \| 0.29* \| 0.41* \| 0.23 \| 0.08* \| 0.17* \| 0.42 \| 0.16 \| 1.00 \| | | | | |
| **Late response** | | | | |
| \|  \| SBP \| DBP \| MCAv \| HR \| O_2_Hb-l \| O_2_Hb-s \| HHb-l \| HHb-s \| \| --- \| --- \| --- \| --- \| --- \| --- \| --- \| --- \| --- \| \| SBP \| 1.00 \|  \|  \|  \|  \|  \|  \|  \| \| DBP \| 0.85* \| 1.00 \|  \|  \|  \|  \|  \|  \| \| MCAv \| 0.12 \| 0.27 \| 1.00 \|  \|  \|  \|  \|  \| \| HR \| 0.04* \| 0.19* \| 0.07 \| 1.00 \|  \|  \|  \|  \| \| O_2_Hb-l \| 0.33 \| 0.36 \| 0.18 \| -0.11 \| 1.00 \|  \|  \|  \| \| O_2_Hb-s \| 0.36 \| 0.34 \| 0.01 \| -0.16 \| 0.60 \| 1.00 \|  \|  \| \| HHb-l \| 0.03 \| 0.03 \| -0.11 \| -0.06 \| 0.00 \| 0.22 \| 1.00 \|  \| \| HHb-s \| 0.12 \| 0.09 \| 0.08 \| -0.12* \| 0.07 \| 0.30 \| 0.30 \| 1.00 \| | | | | |
| **Sit-stand** | |  |  |  |
| **Young (18-35 years) adults (n=11)** | |  |  |  |
| **Initial response** | | | | |
| \|  \| SBP \| DBP \| MCAv \| HR \| O_2_Hb-l \| O_2_Hb-s \| HHb-l \| HHb-s \| \| --- \| --- \| --- \| --- \| --- \| --- \| --- \| --- \| --- \| \| SBP \| 1.00 \|  \|  \|  \|  \|  \|  \|  \| \| DBP \| 0.89 \| 1.00 \|  \|  \|  \|  \|  \|  \| \| MCAv \| 0.29 \| 0.44 \| 1.00 \|  \|  \|  \|  \|  \| \| HR \| -0.80* \| -0.67* \| 0.01 \| 1.00 \|  \|  \|  \|  \| \| O_2_Hb-l \| 0.70 \| 0.65 \| 0.30 \| -0.67* \| 1.00 \|  \|  \|  \| \| O_2_Hb-s \| 0.79* \| 0.81* \| 0.24 \| -0.76* \| 0.85 \| 1.00 \|  \|  \| \| HHb-l \| 0.07 \| 0.16 \| -0.06 \| -0.17 \| -0.07 \| 0.16 \| 1.00 \|  \| \| HHb-s \| 0.60* \| 0.72* \| 0.36 \| -0.41* \| 0.42 \| 0.64 \| 0.21 \| 1.00 \| | | | | |
| **Late response** | | | | |
| \|  \| \| SBP \| \| \| DBP \| \| \| MCAv \| \| \| HR \| \| \| O_2_Hb-l \| \| \| O_2_Hb-s \| \| \| HHb-l \| \| \| HHb-s \| \| \| --- \| --- \| --- \| --- \| --- \| --- \| --- \| --- \| --- \| --- \| --- \| --- \| --- \| --- \| --- \| --- \| --- \| --- \| --- \| --- \| --- \| --- \| --- \| --- \| --- \| \| SBP \| \| 1.00 \| \| \|  \| \| \|  \| \| \|  \| \| \|  \| \| \|  \| \| \|  \| \| \|  \| \| \| DBP \| \| 0.64* \| \| \| 1.00 \| \| \|  \| \| \|  \| \| \|  \| \| \|  \| \| \|  \| \| \|  \| \| \| MCAv \| \| -0.01* \| \| \| 0.19 \| \| \| 1.00 \| \| \|  \| \| \|  \| \| \|  \| \| \|  \| \| \|  \| \| \| HR \| -0.34* \| \| 0.09* \| \| \| 0.07 \| \| \| 1.00 \| \| \|  \| \| \|  \| \| \|  \| \| \|  \| \| \| O_2_Hb-l \| 0.35 \| \| \| 0.36 \| \| \| 0.18 \| \| \| -0.19* \| \| \| 1.00 \| \| \|  \| \| \|  \| \| \|  \| \| \| O_2_Hb-s \| \| 0.26 \| \| \| 0.41 \| \| \| 0.07 \| \| \| -0.03 \| \| \| 0.59 \| \| \| 1.00 \| \| \|  \| \| \|  \| \| \| HHb-l \| \| -0.10 \| \| \| -0.05 \| \| \| -0.22 \| \| \| 0.11 \| \| \| -0.16 \| \| \| 0.12 \| \| \| 1.00 \| \| \|  \| \| \| HHb-s \| 0.06 \| \| \| 0.27* \| \| \| 0.10 \| \| \| 0.13 \| \| \| 0.22 \| \| \| 0.57 \| \| \| 0.31 \| \| \| 1.00 \| \| | | | | |
| **Older (>65 years) adults (n=30)** |  |  |  |  |
| **Initial response** | | | | |
| \|  \| SBP \| DBP \| MCAv \| HR \| O_2_Hb-l \| O_2_Hb-s \| HHb-l \| HHb-s \| \| --- \| --- \| --- \| --- \| --- \| --- \| --- \| --- \| --- \| \| SBP \| 1.00 \|  \|  \|  \|  \|  \|  \|  \| \| DBP \| 0.83 \| 1.00 \|  \|  \|  \|  \|  \|  \| \| MCAv \| 0.09 \| 0.30 \| 1.00 \|  \|  \|  \|  \|  \| \| HR \| -0.28* \| -0.12* \| 0.19 \| 1.00 \|  \|  \|  \|  \| \| O_2_Hb-l \| 0.58 \| 0.60 \| 0.27 \| -0.23* \| 1.00 \|  \|  \|  \| \| O_2_Hb-s \| 0.50* \| 0.53* \| 0.08 \| -0.33* \| 0.82 \| 1.00 \|  \|  \| \| HHb-l \| -0.20 \| -0.09 \| -0.10 \| 0.03 \| 0.00 \| 0.11 \| 1.00 \|  \| \| HHb-s \| 0.15* \| 0.30* \| 0.32 \| 0.10* \| 0.21 \| 0.25 \| 0.18 \| 1.00 \| | | | | |
| **Late response** | | | | |
| \|  \| SBP \| DBP \| MCAv \| HR \| O_2_Hb-l \| O_2_Hb-s \| HHb-l \| HHb-s \| \| --- \| --- \| --- \| --- \| --- \| --- \| --- \| --- \| --- \| \| SBP \| 1.00 \|  \|  \|  \|  \|  \|  \|  \| \| DBP \| 0.84* \| 1.00 \|  \|  \|  \|  \|  \|  \| \| MCAv \| 0.19* \| 0.23 \| 1.00 \|  \|  \|  \|  \|  \| \| HR \| 0.11* \| 0.30 \| 0.05 \| 1.00 \|  \|  \|  \|  \| \| O_2_Hb-l \| 0.33 \| 0.38 \| 0.07 \| 0.06* \| 1.00 \|  \|  \|  \| \| O_2_Hb-s \| 0.28 \| 0.28 \| -0.10 \| 0.11 \| 0.70 \| 1.00 \|  \|  \| \| HHb-l \| -0.14 \| -0.12 \| -0.23 \| -0.01 \| -0.03 \| 0.07 \| 1.00 \|  \| \| HHb-s \| 0.04 \| 0.06* \| 0.01 \| 0.05 \| 0.18 \| 0.38 \| 0.15 \| 1.00 \| | | | | |

*Significantly (p<0.05) different between younger (18-35 years) and older (>65 years) participants.

**SBP: Systolic blood pressure, DBP: diastolic blood pressure, MCAv: mean blood flow velocity in middle cerebral artery, O_2_Hb-l: oxygenated hemoglobin measured with long channels, O_2_Hb-s: oxygenated hemoglobin measured with short channels, HHb-l: deoxygenated hemoglobin measured with long channels, HHb-s: deoxygenated hemoglobin measured with short channels.**

**Fig. s5 Supine-stand responses in older (>65 years) (A, C, E, G) and younger (18-35 years) (B, D, F, H) participants of A+B blood pressure (BP), systolic in red** **(solid line), diastolic in blue** **(dashed line), and heart rate (HR) in green** **(dashed-dotted line), C+D cerebral blood velocity (MCAv), systolic in red (solid line), diastolic in blue (dashed line), E+F cerebral oxygenation measured with long channels, oxygenated hemoglobin in red and deoxygenated hemoglobin in blue (dashed line) and G+H cerebral oxygenation measured with short channels, oxygenated hemoglobin in red and deoxygenated hemoglobin in blue (dashed line). All signals are shown from one minute before standing up to 175 seconds after standing up, averaged over all supine-stand transitions and all older or younger participants. Standing up is indicated by a vertical black dashed line. Shaded areas show standard deviations for all signals.**

**Fig. s6 Sit-stand responses in older (>65 years) (A, C, E, G) and younger (18-35 years) (B, D, F, H) participants of A+B blood pressure (BP), systolic in red (solid line), diastolic in blue (dashed line), and heart rate (HR) in green (dashed-dotted line), C+D cerebral blood velocity (MCAv), systolic in red (solid line), diastolic in blue (dashed line), E+F cerebral oxygenation measured with long channels, oxygenated hemoglobin in red and deoxygenated hemoglobin in blue (dashed line) and G+H cerebral oxygenation measured with short channels, oxygenated hemoglobin in red and deoxygenated hemoglobin in blue (dashed line). All signals are shown from one minute before standing up to 175 seconds after standing up, averaged over all sit-stand transitions and all older or younger participants. Standing up is indicated by a vertical black dashed line. Shaded areas show standard deviations for all signals.**

**References**

1. Scholkmann, F., et al., *How to detect and reduce movement artifacts in near-infrared imaging using moving standard deviation and spline interpolation.* Physiological Measurement, 2010. **31**(5): p. 649-662.
